# Ventral Transorbital Apicoectomy of the Maxillary Second and Third Molar Teeth in Guinea Pigs (*Cavia porcellus*): 26 Cases

**DOI:** 10.3390/vetsci13010053

**Published:** 2026-01-07

**Authors:** Justyna Ignaszak-Dziech, Vladimir Jekl, Tomasz Piasecki

**Affiliations:** 1 Center for the Diagnosis and Treatment of Exotic Animals, Veterinary Clinic “Zwierzyniec”, ul. Bulwar Ikara 31b, 54-130 Wroclaw, Poland; 2 Department of Pharmacology and Pharmacy, Faculty of Veterinary Medicine, University of Veterinary Sciences Brno, Palackeho tr. 1946/1, 61242 Brno, Czech Republic; 3 Jekl & Hauptman Veterinary Clinic, Mojmirovo namesti 3105/6a, 61200 Brno, Czech Republic; 4 Department of Epizootiology and Clinic of Bird and Exotic Animals, Faculty of Veterinary Medicine, University of Environmental and Life Sciences, pl. Grunwaldzki 45, 50-366 Wrocław, Poland

**Keywords:** transorbital, maxilla, apicoectomy, macrodont, abscess, guinea pig, dental disease, malocclusion

## Abstract

A common cause of teeth malocclusion and feeding disorders in guinea pigs is macrodontia and odontogenic abscesses. If the maxillary second or third molar teeth are affected, surgical access to them has so far been achieved through enucleation or orbital evisceration due to their location at the base of the orbit. The study aims to demonstrate a transorbital surgical approach to the apices of the maxillary molar teeth (M2 and M3) in guinea pigs, allowing preservation of the eye. Twenty six apicoectomies of maxillary M2 and M3 were performed. The surgical approach involved a skin incision above the zygomatic arch, followed by soft tissue dissection, incision of the orbital ligament, and gentle dorsolateral displacement of the eyeball. Blunt dissection between the lacrimal and zygomatic glands provided direct access to the affected tooth apices, which were removed using a dental bur. After the procedure, the soft tissues and the eye were repositioned and the skin was sutured. All animals recovered uneventfully. The described method may be applied in cases where it is necessary to perform maxillary second and/or last molar tooth apicoectomy while avoiding damage to the eyeball. Care must be taken to protect the corneal surface of the affected eye.

## Simple Summary

Dental overgrowth and infections are common causes of feeding problems and pain in guinea pigs. When these conditions affect the last two upper teeth, which are located very close to the eye, and until now surgical treatment often required removal of the eye to gain access. The aim of this study was to describe and evaluate a new surgical approach that allows treatment of the affected tooth and the alveolus while preserving the eye. In this study, 26 procedures were performed in guinea pigs using a surgical access through the eye socket, carefully moving the eye aside without damaging it. This approach allowed direct access to the diseased tooth and its socket, which were removed using dental instruments, after which the eye and surrounding tissues were returned to their normal position. All animals recovered well without complications, and the eye was preserved in every case. This method provides a less invasive alternative to eye removal and may improve both the welfare and quality of life of guinea pigs suffering from severe dental disease.

## 1. Introduction

Apicoectomy is defined as the removal of the root tip/apex of a brachydont tooth and is used in small animal, canine and feline dentistry when conventional root canal treatment cannot completely cure a periapical infection. After creating an external access, the alveolar socket is cleaned and the root tip is removed with a dental drill, while the crown and the main part of the root remain in the socket [1,2,3].

In rodents, such as the guinea pig (*Cavia porcellus*), it is not possible to speak of the removal of the root tip. The dentition of guinea pigs is hypsodont and elodont, i.e., they have long crowns, grow continuously throughout their lives and are subject to constant wear of the occlusal surface [4,5,6,7,8,9]. Furthermore, all teeth are aradicular and have an open apex containing germ cells and an anatomical crown comprising the reserve and clinical crowns. The pulp cavity, which is also filled with germinal tissue, extends from the apex towards the occlusal surface and into the crown. Each incisor of a guinea pig has a single pulp cavity, whereas each premolar and each molar has two pulp cavities [4,5]. Therefore, in rodents, the apical part of the tooth crown is removed during apicoectomy. Using a dental bur, an osteotomy of the alveolar bone is performed to expose the apical portion of the crown. Subsequently, the dental pulp and the apical part of the crown are removed with the bur until bleeding ceases and a transverse cross-section of the tooth crown is obtained. After the procedure is performed and the germinal cells are removed, the deposition of new tooth substance (tooth growth) ceases. However, the tooth crown continues to move towards the occlusal surface/gingivally due to the action of the periodontal ligaments, and the tooth is still subject to continuous wear of the occlusal surface [9]. As the remainder of the tooth crown stays in the alveolar socket, healing processes take place so that the apical part of the socket is already filled with healing tissue when a small protruding crown fragment is extracted.

Apicoectomy can be performed both in sterile conditions to halt further tooth growth and also when tooth inflammation is suspected. In guinea pigs, apicoectomy is most often performed in cases of macrodontia or odontogenic abscesses. Macrodont teeth are, by definition, larger than normal teeth. Structural changes have been observed in the crown and apex of macrodont teeth in guinea pigs [5]. These changes most commonly occur in the maxillary and mandibular second and third molars [5,6,7]. The enlargement of the tooth circumference in guinea pigs leads to malocclusion and feeding difficulties, making them frequent dental patients. A similar situation occurs with periapical inflammation of these teeth, which causes significant pain at the base of the orbit and contributes to feeding difficulties and exophthalmos.

A search of databases Medline, Web of Knowledge, and Scince Direct with the string “guinea pig” AND “apicoectomy” OR “apicectomy” OR “macrodontia” OR “abscess” on 18 February 2025, and review of 3 recent textbooks [3,10,11] described the surgical access to the maxillary second and third molar teeth, which was associated with enucleation or evisceration. Although effective in providing exposure to the affected teeth, these techniques necessitate removal of the eyeball, which, in many cases, is not primarily diseased and may remain anatomically and functionally normal. Enucleation or evisceration represents a highly invasive procedure associated with irreversible loss of vision and significant perioperative trauma. This study aims to demonstrate a novel surgical access to the maxillary second and third molar apices in guinea pigs via a ventral transorbital approach, allowing apicoectomy without enucleation. Ventral transorbital approach provides direct access to the affected tooth apices through the orbit without the need for enucleation, thereby reducing surgical trauma and enabling treatment of dental disease while maintaining ocular integrity.

## 2. Materials and Methods

### 2.1. Case Selection, Inclusion Criteria

The prospective study includes only client-owned guinea pigs that underwent apicoectomy of the maxillary M2 or M3 without the need for enucleation or evisceration, both during the procedure and in the recovery period. All procedures were performed for therapeutic purposes and involved animals belonging to private owners who consented to the procedures described in this paper and to the use of the results for publishing purposes. The procedures were performed at two institutions: by JID at the Centre for Diagnosis and Treatment of Exotic Animals, Zwierzyniec Veterinary Clinic, Wrocław, Poland, and by VJ at the Jekl & Hauptman Veterinary Clinic, Brno, Czech Republic. The study period ran from January 2023 to January 2024.

Patients were presented with anorexia, weight loss, defecation disorders (soft faeces) or unilateral exophthalmos. All guinea pigs underwent a general physical examination, including oral cavity examination, urinalysis, plasma chemistry and haematology analyses, and computed tomography (CBCT, cone beam computed tomography) prior to the surgery. The detailed dental examination with a rigid endoscope, blood sampling, and the computed tomography of the head were performed under general anaesthesia (intramuscular premedication with 0.5 mg/kg midazolam, 0.3–0.5 mg/kg butorphanol with anaesthesia maintained 1–3% isoflurane, or with alfaxalone 1 mg/kg and butorphanol 0.1 mg/kg with anaesthesia maintained 1–3% isoflurane).

Inflammation of the tooth crown or alveolar socket was suspected in cases where the CBCT examination showed the presence of hypodense areas of the crown (lysis), new bone formation, alveolar bone resorption and/or widening of the periodontal space. Both macrodont teeth and those without typical structural changes in macrodontia were assessed for these conditions. Patients with macrodont teeth without visible signs of inflammation were qualified for the procedure due to generalised malocclusion. In this situation, the intervals between dental corrections were shorter than three weeks, there was no full improvement in appetite despite the dental trimming, and mucosal injury around the macrodont tooth persisted.

Indication for surgery required the presence of the above-described CBCT findings combined with either an intact globe or mild to severe keratoconjunctivitis and ocular surface dryness, provided that the globe was considered salvageable and had a favourable prognosis for recovery following elimination of the primary cause. Patients presenting with globe prolapse or extensive, non-salvageable ocular damage were excluded from the described surgical protocol.

### 2.2. Ocular Disease

**Figure 1:**
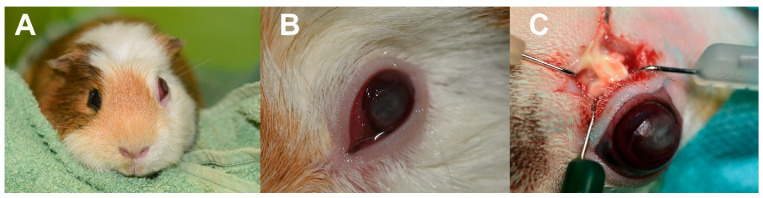
Right eye exophthalmos in a guinea pig associated with apical maxillary molar infection in a guinea pig (**A**–**C**). (**A**) obvious left-sided exophthalmos, (**B**) detailed view of the affected eye with keratoconjunctivitis and dry corneal surface, (**C**) perioperative view of the incision and presence of pus. (Courtesy of Dr. Vladimir Jekl).

The cornea and conjunctiva of all operated animals without odontogenic abscesses were healthy before surgery. All animals with odontogenic abscesses had mild to severe keratoconjunctivitis associated with exophthalmos and dry eye (Figure 1A,B). The data on age, sex, teeth operated on and indications for the individual procedures are summarised in Table 1.

### 2.3. Surgical Procedure

The patient’s state of health was stabilised before the planned operation. The surgical procedures were performed by 1 of the authors (JID or VJ), both of whom are experienced surgeons; in both instances, the procedures were conducted in a standardised manner according to the planned surgical protocol. General anaesthesia was induced using two different protocols depending on the referral centre. In the first centre (Centre for Diagnosis and Treatment of Exotic Animals, Zwierzyniec Veterinary Clinic, Wrocław, Poland), anaesthesia was induced with a combination of butorphanol (0.3–0.5 mg/kg), medetomidine (0.05–0.1 mg/kg) and ketamine (10–15 mg/kg) administered intramuscularly. As additional analgesia, meloxicam (0.5 mg/kg) was administered at least one hour before the procedure. In the second centre (Jekl & Hauptman Veterinary Clinic, Brno, Czech Republic), an alternative anaesthesia protocol included a combination of metamizole (50 mg/kg), buprenorphine (0.03 mg/kg), midazolam (0.1–0.2 mg/kg), medetomidine (0.02–0.04 mg/kg) and ketamine (3–10 mg/kg) administered intramuscularly. In both centres, anaesthesia was maintained with isoflurane via a face/nasal mask with or without ketamine infusion at a continuous rate (0.3 mg/kg/h). Anaesthesia monitoring included respiratory rhythm and depth monitoring, thoracic auscultation, ECG and SpO_2_ measurement. Isotonic fluid therapy (10 mL/kg) was administered perioperatively via continuous infusion. Retrospective analysis of the case histories revealed no influence of the anaesthetic protocol or peri-anaesthetic course on the subsequent treatment outcome.

The patient was positioned laterally with the rostral part of the head supported and straps were used to stabilise the position to ensure that the eyeball was perpendicular to the surgical table. The surgical site was prepared as standard, and the eyes were protected with ophthalmic ointment or gel.

**Figure 2:**
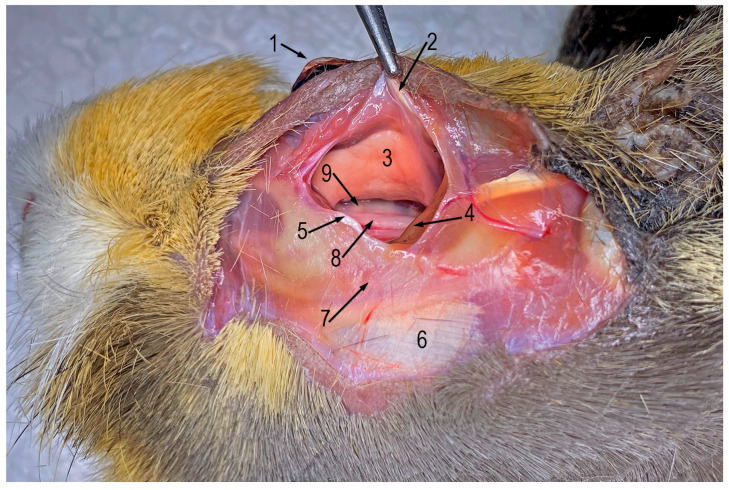
Orbital region on an anatomical specimen. 1—eyeball, 2—orbital ligament, 3—lacrimal gland, 4—zygomatic gland, 5—fascia, 6—masseter muscle, 7—zygomatic arch, 8—maxillary nerve, 9—M2 maxillary tooth socket. (Courtesy of Dr. Justyna Ignaszak—Dziech and Dr. Tomasz Piasecki).

**Figure 3:**
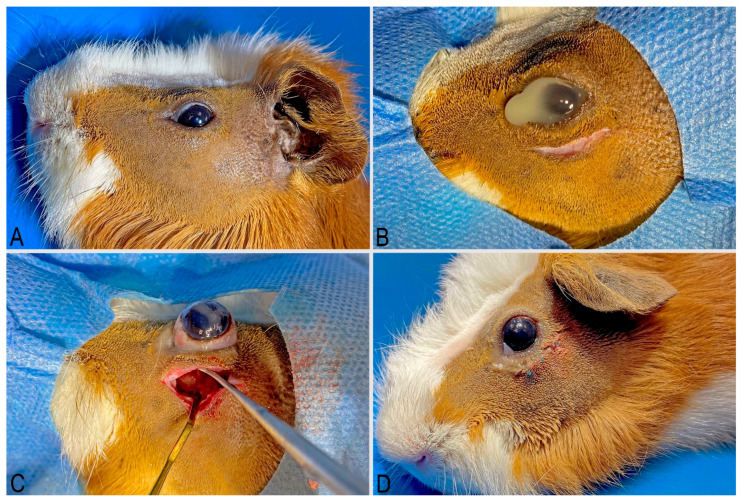
Stages of an apicoectomy procedure via an orbital approach. (**A**) preparation of the surgical field in the orbital region. (**B**) eyeball protected with ophthalmic ointment. Skin incision made above the zygomatic arch. (**C**) eyeball displaced from the orbit, exposing the apex of the M2 maxillary tooth after retracting the lacrimal and zygomatic glands with instruments. (**D**) postoperative wound sutured with a single stitch, and the eyeball returned to the orbit. (Courtesy of Dr. Justyna Ignaszak—Dziech and Dr. Tomasz Piasecki).

The surgical field and procedure were described using terminology consistent with *Nomina Anatomica Veterinaria* [12]. The operative field included the periocular area (Figure 2 and Figure 3A). The skin incision was made just above the zygomatic arch, at the level of the orbicularis oculi muscle (Figure 3B). The orbicularis oculi muscle was sharply dissected from the zygomatic bone, towards the direction of the orbital ligament. The rostral fragment of the orbital ligament was sharply removed. A Lone-star retractor was then attached to the wound to allow better operative view.

If the procedure was performed due to macrodontia of M2 or M3 without signs of inflammation, the eyeball was gently displaced from the orbit and the space between the lacrimal and zygomatic glands was bluntly dissected. Once the orbital floor was reached and the apex of the tooth was exposed, the periosteum was retracted from the alveolar bone using a periosteal elevator. A flat spatula was used to retract the lacrimal gland and eyeball dorsally (Figure 3C). Intraoperatively, the position of the spatula and the globe was stabilised by a surgical assistant. The osteotomy of the alveolar bone was performed with a round burr on a dental micromotor handpiece (30,000 rpm). After reaching the apex of the affected tooth, drilling was continued to complete the apicoectomy until bleeding from the crown subsided and a complete transverse cross-section of the tooth crown was obtained. After complete apicoectomy, the surgical field was debrided with cotton tips, sterile saline solution and surgical suction. The instruments were withdrawn, and the eyeball was returned to the orbit. The skin was sutured with a simple interrupted pattern using non-absorbable 4–5/0 suture material (Dafilon^®^, B. Braun, Melsungen, Germany) (Figure 3D). In the case of odontogenic abscesses, the wound was debrided with a bone curette and cotton-tips (Figure 1C). The alveolar osteotomy was performed as described above. Samples were taken for bacteriological analysis and an antibiogram was performed. After thorough debridement, the wound was marsupialized.

**Figure 4:**
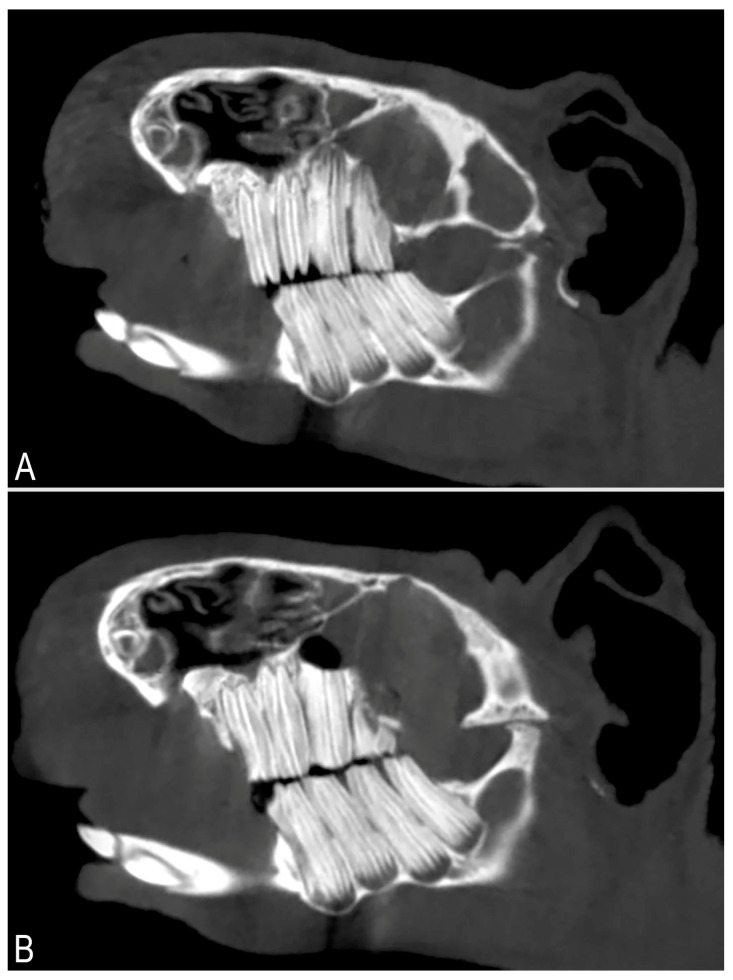
Tomograms of a guinea pig’s skull in the sagittal planes, showing the left maxillary cheek teeth. (**A**) structural change typical of macrodontia visible in the mesial part of the M2 left maxillary tooth. Narrowed mesial pulp chamber, with the apex and distal pulp chamber normal. (**B**) hypodense area at the apex of the M2 left maxillary tooth indicates a properly performed apicoectomy. (Courtesy of Dr. Justyna Ignaszak—Dziech and Dr. Tomasz Piasecki).

A perioperative CBCT examination was performed after the procedure (Figure 4). A successful apicoectomy was only observed in cases where the entire apical part of the tooth was removed.

### 2.4. Postoperative Care and Follow-Up Data

Postoperative care included supportive care, fluid therapy and, if necessary, syringe feeding. Analgesia included an opioid (tramadol 10 mg/kg orally q12h) and NSAIDs (meloxicam 1 mg/kg orally q12h or metamizole 50 mg/kg, orally q8 or 12h), with optional gabapentin (25 mg/kg orally q12h). Buprenorphine (0.03 mg/kg intramuscularly q8h) was only administered in a short postoperative phase while the animal was hospitalised. Analgesic drugs were administered for at least 10–14 days. To protect the eyes, ophthalmological preparations such as hyaluronic acid and ofloxacin (only for keratitis and conjunctivitis) were administered in the form of ointments and drops for 10–14 days as required. In addition, systemic antibiotic therapy was administered: long-acting oxytetracycline (25 mg/kg subcutaneously q72h) or sulfamethoxazole + trimethoprim (25 mg/kg orally q12h) for 14 days to limit bacterial inflammation of the alveolus, ocular tissue and orbit. For bacterial abscesses, doxycycline (5 mg/kg orally q12h) and metronidazole (25 mg/kg orally q12h) were the antibiotics of first choice, which were then adjusted according to the results of bacteriology and antimicrobial susceptibility testing.

In the case of an apicoectomy on a macrodontic tooth without signs of inflammation, the sutures were removed 14 days after the procedure. If the procedure involved marsupialisation of an abscess, the wounds were checked on the 3rd, 7th and 14th day after the procedure. On day 14, the stitches were removed and the wound was healed by secondary intention. Radiological checks of the tooth after apicoectomy were performed under general anaesthesia 30, 60 and possibly 100 days after the procedure. Regular dental corrections were performed as required.

## 3. Results

In 2023–2024, the apicoectomy was performed via the ventral transorbital approach in 26 privately kept guinea pigs (8 females and 18 males) aged between 1.5 and 6 years (mean 3.5 years). A total of 27 maxillary molars underwent apicoectomy (19 M2 and 8 M3); in one case, both M2 and M3 were operated on in a single procedure. Fourteen of the 27 teeth were macrodont teeth, and in 9 of these 14 cases, an inflammation of the crown or tooth socket was suspected. The remaining 13 teeth showed no signs of macrodontia and underwent apicoectomy due to inflammation of the crown or tooth socket.

General anaesthesia was uneventful in all cases, with no peri-anaesthetic complications observed and all animals recovered fully without neurological or systemic sequelae. Apicoectomy was successfully performed in all cases, as confirmed by postoperative radiographic or CT examination. In all patients, the globe was preserved and maintained functional integrity following completion of the recovery period. All operated patients survived the postoperative recovery period until the removal of the crown after apicoectomy. The postoperative wound healed properly in all patients and the socket healed completely without complications. The operated tooth crown was extracted or exfoliated within 2 to 4 months. No differences were observed in the time to crown removal between maxillary second (M2) and third (M3) molars; however, teeth affected by macrodontia required a longer interval prior to removal, most commonly closer to 4 months, whereas teeth with inflammatory changes without features of macrodontia were more frequently removed approximately 2 months after the procedure.

In patients diagnosed with macrodontia without signs of inflammation and without corneal damage prior to the procedure, tarsorrhaphy was not performed immediately after the procedure. No eye complications were observed in 8/14 patients (57%). Mild ocular complications, which included superficial corneal injury and mild conjunctival swelling, occurred in 3/14 (21%) patients but did not require a change in pharmacological treatment. In both cases, without complications and those with mild complications, the standard treatment regimen included 0.4% hyaluronic acid gel applied 4–5 times daily and ofloxacin ointment applied 3 times daily for 7–10 days. In severe complications (3/14 patients (21%)), such as extensive corneal injuries with stromal oedema and severe conjunctival swelling, a complete temporary tarsorrhaphy was also performed for 3–5 days. In this case, the continuation of local ophthalmological preparations was discontinued. The treatment lasted 10–14 days in these cases.

In the patients with odontogenic abscesses, mild to severe keratoconjunctivitis was observed in all animals during the clinical examination. Postoperative treatment included dexpanthenol gel (Recugel eye gel, Baush & Lomb, Bridgewater, NJ, USA) applied 5–6 times daily for 7–20 days and ofloxacin drops applied 3 times daily for 7–10 days. The conjunctiva and cornea healed within 5–21 days after the procedure. In 2 guinea pigs, wound healing was unsatisfactory due to the presence of inflamed bone sequestra, and these patients had to undergo surgery on the 5th and 7th day. After the second procedure, the wound was completely healed within the next 7–14 days.

## 4. Discussion

Dental disease is a common reason why guinea pigs visit an exotic animal veterinarian in daily practise. In a study by Minarikova et al. (2015) [13], the incidence of dental disease was 36.3% (363 animals out of 1000 guinea pigs). Patients are usually presented with reduced appetite, production of small faecal pellets or weight loss [11,14]. A common cause of malocclusion in guinea pigs is periapical inflammation or macrodontia [15]. These pathologies often affect the last two molars in both the upper and lower jaw [6,7]. The use of computed tomography in the diagnosis of dental diseases enables the detection of inflammatory and structural dental alterations at an early stage, allowing earlier surgical intervention [15,16,17]. This is particularly true for pathological changes in the second and third maxillary molars of the guinea pig, whose apices are located in the ventral part of the orbit. Both inflammation with abscess formation and macrodontia in advanced stages lead to exophthalmos and secondary corneal damage, which is also associated with severe pain. If these changes are not diagnosed in time, enucleation may be necessary. In periapical abscesses, the only effective treatment is extraction of the inflamed tooth and debridement of the abscess capsule and alveolus [17]. The affected tooth can be extracted or an apicoectomy can be performed [17]. To date, apicoectomy has been sporadically mentioned for the treatment of dental disease in guinea pigs [17,18,19]. Therefore, this is the first study to describe the use of apicoectomy for both periapical abscesses and malocclusion due to macrodontia. In addition, this study describes an osteotomic approach to the apices of the second and third upper molars, which allows the eyeball to be preserved.

To correctly perform an apicoectomy on a hypsodont and elodont tooth, the entire apical part of the crown must be removed and the pulp cavity cleaned of germinal cells. It should be noted that each premolar and molar tooth of a guinea pig has two pulp cavities, and each of them must be thoroughly cleaned. Inflammation of the alveolar socket often results in resorption of the apical part of the crown and the alveolar bone. Therefore, there is no significant difficulty in effectively removing the growth zone of the tooth. Similarly, in macrodont teeth, the structural remodelling process begins at the apex of the tooth, with hard tissue filling the pulp cavities and significantly reducing the pulpal cavity [5]. Thus, structural remodelling also facilitates proper apicoectomy, especially since both the crown and the apex of the tooth are enlarged and thus more surgically accessible. In the authors’ experience, despite the interruption of tooth growth after apicoectomy, the crown moves towards the occlusion and continues to wear down, leaving a healed alveolar socket. This is possible due to the activity of the dentoalveolar ligaments, which constantly change their position and move the crown coronally/intraorally [13]. Incorrectly performed apicoectomy, in which germinal cells remain in the apex or pulp cavity, can lead to partial regrowth of the crown, although complete regrowth of the crown is rare [18,19,20]. Such situations are likely to lead to complications during alveolar healing. In odontogenic abscesses with pus fistulae into the orbital space, surgical access is easier in the authors’ experience, as the pus pushes the ocular structures and the lacrimal glands away from each other, allowing the surgeon to reach the affected apex directly after careful wound debridement and pus removal.

Corneal damage is one of the most common complications of craniofacial surgery. In humans undergoing general anaesthesia for non–ocular surgery, the incidence of corneal damage can be as high as 44%, while in dogs it ranges from 1.9% to 19.1% [21,22,23,24,25]. Although the procedure described in our study does not directly affect the eyeball, the risk of ocular complications is high due to the manipulation of the orbit. The incidence of corneal abrasions as a result of the procedure was 23%. Most complications are due to corneal drying caused by incomplete eyelid closure during anaesthesia [23,24]. To protect the eyeball, an intraoperative tarsorrhaphy could be performed [24]. However, as the eyeball has to be lifted and moved out of the orbit during the described procedure, this is not recommended. Tear production decreases during anaesthesia, which contributes to drying of the corneal surface [23,24,25]. To avoid such complications, eye ointments should be applied to the surface of the eyeball during the procedure. Ointments remain on the corneal surface longer than drops and offer better protection [24,26,27]. Due to direct contact with the lacrimal gland and zygomatic gland during the procedure, there is a risk of postoperative swelling of these structures, which may lead to exophthalmos after the procedure and further expose the cornea to desiccation. Both the anaesthetics used during anaesthesia [24] and the pressure from gland swelling disrupt the blood flow in the orbit, which can lead to corneal oedema. To mitigate such complications, non-steroidal (e.g., meloxicam) or steroidal (e.g., dexamethasone) anti-inflammatory drugs should be used during the recovery period [26,27]. In odontogenic abscesses, the exophthalmic eye usually already shows corneal damage and keratitis due to the dry eye, so that the healing of the cornea is accelerated as soon as the eye is brought back into the correct position. Another risk factor is manipulation with instruments in close proximity to the eyeball [23,24]. In the surgical field, instruments should be used with extreme caution and both the surgical drape and the retractor should not touch the eyeball.

Root apex resection plays an important role in the treatment of dental disease in guinea pigs due to periapical infection and macrodontia. It allows removal of the affected tooth in a much less traumatic way than conventional intraoral extraction and ensures better healing of the alveolus. The described approach also enables the preservation of the socket, which is crucial if it has not been damaged by changes to the tooth tips. This procedure is applicable to both periapical abscesses and non-inflammatory changes (i.e., macrodontia). Due to the high exposure of the eyeball during the procedure and the manipulations within the orbit, adequate protection of the eyeball is required during the procedure and in the postoperative period. In the authors’ experience, the opposing mandibular molars must be monitored for clinical crown lengthening, especially in cases where two maxillary molars have been operated on. In such cases, regular dental trimming may be necessary, or apicoectomy of the opposing molar teeth should be considered.

## Figures and Tables

**Table 1:** The table provides information on the age, gender of each patient, and the indications for performing apicoectomy on the listed teeth. F—female, M—male, Fc—spayed female, Mc—neutered male, L—left maxilla, R—right maxilla.

Lp.	Age at the Time of Surgery	Sex	Operated Tooth	Indications for the Procedure
1.	2.5	M	M3 L	Macrodontia, suspected inflammation of the tooth and alveolus
2.	3.5	F	M2 L	Macrodontia
3.	2.5	M	M2 R	Macrodontia, suspected inflammation of the alveolus
4.	2.5	M	M3 R	Macrodontia, suspected inflammation of the alveolus
5.	5.5	M	M2 L	Macrodontia
6.	2.5	F	M2 L	Macrodontia, suspected inflammation of the tooth and alveolus
7.	2	M	M2 L	Macrodontia, suspected inflammation of the tooth and alveolus
8.	4	M	M2 L	Macrodontia
9.	2	M	M2 R	Macrodontia, suspected inflammation of the tooth and alveolus
10.	5.5	F	M2 L	Macrodontia
11.	2.5	M	M2 L	Macrodontia
12.	3.5	M	M3 L	Macrodontia, suspected inflammation of the tooth and alveolus
13.	3.5	M	M2 L	Macrodontia, suspected inflammation of the tooth and alveolus
14.	5.5	M	M2 L	Suspected inflammation of the tooth and alveolus, periapical abscess
15.	3.5	Mc	M2 R	Suspected inflammation of the tooth and alveolus, periapical abscess
16.	3	Mc	M2 R	Suspected inflammation of the tooth and alveolus, periapical abscess
17.	5.5	Fc	M3 L	Suspected inflammation of the tooth and alveolus, periapical abscess
18.	3.5	Fc	M2 L	Suspected inflammation of the tooth and alveolus, periapical abscess
19.	5	F	M2 L	Suspected inflammation of the tooth and alveolus, periapical abscess
20.	2.5	M	M2 L	Macrodontia, suspected inflammation of the tooth and alveolus
21.	2	Mc	M2 R	Suspected inflammation of the tooth and alveolus, periapical abscess
22.	4.5	M	M2, M3 R	Suspected inflammation of the tooth and alveolus, periapical abscess
23.	3.5	M	M3 L	Suspected inflammation of the tooth and alveolus, periapical abscess
24.	6	Fc	M2 L	Suspected inflammation of the tooth and alveolus, periapical abscess
25.	5	Fc	M3 L	Suspected inflammation of the tooth and alveolus, periapical abscess
26.	1.5	M	M3 L	Suspected inflammation of the tooth and alveolus, periapical abscess

## Data Availability

The original contributions presented in this study are included in the article. Further inquiries can be directed to the corresponding authors.
